# Seasonal variation in prevalence of *Borrelia burgdorferi* sensu lato and *Neoehrlichia mikurensis* in *Ixodes ricinus* nymphs in southern Norway

**DOI:** 10.1186/s13028-026-00860-x

**Published:** 2026-04-15

**Authors:** Andrea Cotes-Perdomo, Arnulf Soleng, Kenedith Méndez-Gutierrez, Kristian Alfsnes, Hans Renssen, Vivian Kjelland, Rose Vikse, Åshild Andreassen, Andrew Jenkins

**Affiliations:** 1 Department of Natural Science and Environmental Health, University of South-Eastern Norway, Bø, Norway; 2 Department of Virology, Norwegian Institute of Public Health, Oslo, Norway; 3 Department of Pest Control, Norwegian Institute of Public Health, Oslo, Norway; 4 Faculty of Basic Sciences, University of Magdalena, Santa Marta, Colombia; 5 Department of Bacteriology, Norwegian Institute of Public Health, Oslo, Norway; 6 Department of Natural Sciences, University of Agder, Kristiansand, Norway

**Keywords:** *Borrelia afzelii*, *Borrelia burgdorferi* s. s., *Borrelia garinii*, Density of infected nymphs, Infection risk

## Abstract

**Background:**

*Borrelia burgdorferi* sensu lato (s. l.) and *Neoehrlichia mikurensis* are the most prevalent pathogenic bacteria found in *Ixodes ricinus* in Norway. Here we evaluate the prevalence of both bacteria and the density of infected ticks, key factors to assess the human infection risk. The study was performed in one location in southern Norway.

**Results:**

An overall prevalence of 12% for *B. burgdorferi* s. l. and 8% for *N. mikurensis* was found. The most prevalent *B. burgdorferi* s. l. genospecies was *Borrelia afzelii*, followed by *Borrelia burgdorferi* sensu stricto and *Borrelia garinii.* The highest density of infected nymphs with *B. burgdorferi* s. l. was in May and the lowest was in November. In the case of *N. mikurensis*, the highest density of infected nymphs was found in September and the lowest in November. *B. burgdorferi* s. l. prevalence was negatively correlated with tick density.

**Conclusions:**

Our results highlight the importance of assessing not only pathogen prevalences but also tick population densities. Extended periods of surveillance in questing ticks are necessary to understand the intricate dynamics of ticks and tick-borne pathogens.

## Background

The castor bean tick *Ixodes ricinus* is the main vector of tick-borne pathogens in Europe [1]. *I. ricinus* uses a broad range of vertebrates as hosts including mammals, birds, and reptiles. Rodents have a crucial epidemiological role in the maintenance of *Borrelia burgdorferi* sensu lato genospecies and *Neoehrlichia mikurensis* [2, 3], the two most prevalent tick-borne pathogens in ticks in Norway [4–6].

Members of the *B. burgdorferi* complex are the causative agents of Lyme borreliosis (LB), with arthritic, neurological, cardiological, or rheumatic manifestations, depending on the genospecies [7]. Despite being the most common human tick-borne infection in the world, the diagnostics and epidemiology of LB continue to pose significant challenges [8, 9]. In Norway, laboratory confirmed cases of LB are notifiable where symptoms of invasive disease are present [10]. The most common genospecies found in Norway of the *B. burgdorferi* complex is *B. afzelii*, followed by *B. garinii*, *B. burgdorferi* sensu stricto, and *B. valaisiana* [11].

*Neoehrlichia mikurensis* is a recently described emerging human pathogen causing neoehrlichiosis [12, 13]. It has been detected in blood samples of patients with erythema migrans and immunosuppressed patients in Norway, but it is not routinely diagnosed nor notifiable. The first confirmed case in the country was reported in 2017 [14–16]. Immunocompromised patients may present with fever, myalgia, arthralgia, rashes or erythema, and vascular complications, while symptoms in immunocompetent patients are usually subclinical [17].

*Ixodes ricinus* ticks infected with *B. burgdorferi* s. l. are abundant throughout the tick’s geographical range in coastal and lowland areas of Norway south of the Arctic circle [18, 19]. The prevalences vary between 4 and 31% [6, 11, 20–22]. The distribution of *N. mikurensis* infected ticks is similar, with prevalences ranging from 6 to 28%, except at parts of the southwestern coast, where it is scarce [5].

Currently, the assessment of prevalence variations at the local level is necessary to evaluate the infection risk [23]. The density of infected nymphs (DIN) is one of the most important criteria to evaluate risk and prevent human infections and it is obtained by assessing pathogen prevalence and vector density [24]. Since ticks spend most of their lifetime off host [25], their questing behavior, feeding, molting, reproductive success and survival are affected by macro- and microclimatic conditions, especially humidity and temperature [1, 26]. This makes it necessary to evaluate variations of prevalence both within and between seasons.

The aim of this study was to investigate the seasonal variation in prevalence of *B. burgdorferi* s. l. and *N. mikurensis* in *I. ricinus* nymphs from Southern Norway throughout the months of tick activity and compare it against tick density. This contributes important knowledge in tick-borne pathogens prevalences and human infection risk.

## Methods

### Collection of ticks

Nymphal ticks were collected at Kilen, Mandal (58°00’N; 07°30’E) in southern Norway, between April and November 2020. No sampling could be conducted in October, due to persistent rain and wet conditions. This site has been regularly monitored for tick-borne encephalitis virus (TBEV) since 2008. Surveillance began after a patient identified this high-tick-density area as the location of their tick bite. The sampling area, chosen as it represents a typical tick habitat in southern Norway, is a small north-facing hillside with an elevation 20–50 m above the sea level and 50–150 m from the sea. The vegetation is mixed forest, dominated by deciduous, broadleaved trees. The undergrowth consists of bilberry (*Vaccinium myrtilus*) shrubs, ferns and grass. Roe deer (*Capreolus capreolus*), as well as high numbers of *Apodemus* spp. and *Myodes* spp. mice were observed during the sampling period. In addition, numerous rodent burrows, bedding sites and tracks created by roe deer were observed.

Tick density was assessed as previously described [27], with an increased number of transects, by dragging a woolen flannel cloth (1 × 1 m with lead weight on the posterior side) along eight 50 m transects for a total of 400 m. Ticks were counted at the end of each transect. The density of nymphs in 100 m^2^ (DON) was calculated from the total of ticks collected in the eight 50 m transects. Following the transects dragging, tick collection was done with the same woolen flannel cloth but without a lead weight as previously described [11]. A minimum of 740 nymphs were collected for TBEV surveillance, and 200 nymphs were collected for *B. burgdorferi* s. l. and *N. mikurensis* detection when possible. The vegetation was dry at all samplings. Ticks were removed from the cloth into tubes, kept on ice during transportation to the laboratory, and stored at -80 °C for later analysis. Adults and larvae were not included in this study.

### DNA extraction

DNA was extracted from individual nymphal ticks. Each tick was prewashed, first with 70% ethanol, then with distilled water. Ticks were quadrisected using flame-sterilized scalpels, and DNA was extracted with the DNeasy Blood and Tissue kit (Qiagen, Hilden, Germany) following the manufacturer’s instructions with an extended lysis step of 16 h at 56 °C.

### Identification of tick species by real-time PCR

For tick species identification, a real-time PCR, amplifying a specific fragment of the *5.8S* gene of *I. ricinus* was carried out to test DNA quality and confirm ticks’ species identity as *Ixodes ricinus*. The real-time PCR used Power Up™ SYBR™ Green Master Mix (1x) (Applied Biosystems, Oslo, Norway), 600 nM of each primer and 5 µL of sample DNA in a total reaction of 25 µL (Tables 1 and 2).

**Table 1:** PCR protocol for the amplification of the *5.8S* gene of *Ixodes ricinus*

		Temp. °C	Time
Pre-cycling stage	Step 1	50	2 min
	Step 2	95	10 min
Cycling stage (45 cycles)	Step 1	95	15 s
	Step 2	60	1 min
Melt curve stage	Step 1	95	15 s
	Step 2	60	1 min
	Step 3	60–95, 0.3 °C increments	
Holding stage	–	15	1 min

**Table 2:** Sequences of primers and probes used in real-time PCRs to confirm the taxonomical identity of *Ixodes ricinus* nymphs, to detect *Neoehrlichia mikurensis* and to identify *Borrelia burgdorferi* sensu lato genospecies

Species	Gene (bp)	Primer/ probe	Sequence (5'-3')	References
*I. ricinus*	*5.8S* (150)	IxriF	GGAAATCCCGTCGCACG	[28]
		IxriR	CAAACGCGCCAACGAAC	
*N. mikurensis*	*groEL* (128)	Neo2F	GCAAATGGAGATAAAAACATAGGTAG	[29]
		Neo2R	CATACCGTCAGTTTTTTCAACTTCTAA	
*B. burgdorferi* s. l	*flaB* (108)	FlaBF	TCAAGAAATAATGSTATTAATGCTGCTAA	[30]
		FlaBR	CCAGCAGCATCATCAGAAGCT	
		*FlaBmA	TGTATCCACTAGAAAGCTT	
	*flaB* (482)	FLA1	AGAGCAACT TACAGACGAAAT TAAT	[31]
		FLA2	CAAGTCTATTTT GGAAAGCACCTAA	

*F:* forward, *R:* Reverse; *TaqMan MGB probe

### *Detection of Borrelia burgdorferi *sensu lato* and Neoehrlichia mikurensis by real-time PCR*

To detect *B. burgdorferi* s. l., a fragment of the *flaB* gene was amplified as previously described [30]. For the detection of *N. mikurensis,* a real-time PCR was performed by amplifying a fragment of the g*roEL* gene; a spiked-in replicate containing 6 × 10^5^ copies of plasmid pNeo for each sample was included to evaluate PCR inhibition (plasmid, primers and PCR protocol described in [29]. See Table 2 for primer and probe sequences.

### *Borrelia burgdorferi *sensu lato* genospecies identification*

A conventional PCR was performed to amplify a 482 bp fragment of the *fla* gene of *B. burgdorferi* species complex as previously described [31]. The PCR conditions were as follows: 3 min at 95 °C and 35 cycles of 30 s at 94 °C, 45 s at 54 °C and 45 s at 72 °C; including 0.1 U/µL of Taq polymerase (Thermo Scientific, Vilnius, Lithuania), 1 × of PCR buffer (Thermo Scientific, Vilnius, Lithuania), 2 mM of MgCl (Thermo Scientific, Vilnius, Lithuania), 0.4 mM of dNTPs and 0.4 pmol of each primer (Table 2). The nucleotide sequences were obtained by Sanger-sequencing of positive PCR products in reverse and forward directions, using the BigDye v3.1 kit on a ABI3500 sequencer (Applied Biosystems™). The sequences were assembled and aligned in Geneious Prime® 2024.0.7. Species identification was done by BLAST search (https://blast.ncbi.nlm.nih.gov/Blast.cgi).

### Statistical analysis

Confidence intervals for prevalence values were calculated with the binconf function of the *Hmisc* package [32]. The number of infected nymphs per 100 m^2^ (density of infected nymphs; DIN) was calculated as a product of each pathogen prevalence and density of nymphs (DON). To evaluate the influence of the prevalence of each pathogen and the DON on the DIN, a Pearson’s correlation test was performed on monthly DON, DIN and prevalence of *B. burgdorferi* s. l. and *N. mikurensis*. *P*-values < 0.05 were considered significant. All the analyses and plots were done in R version 4.3.2.

## Results

**Fig. 1:**
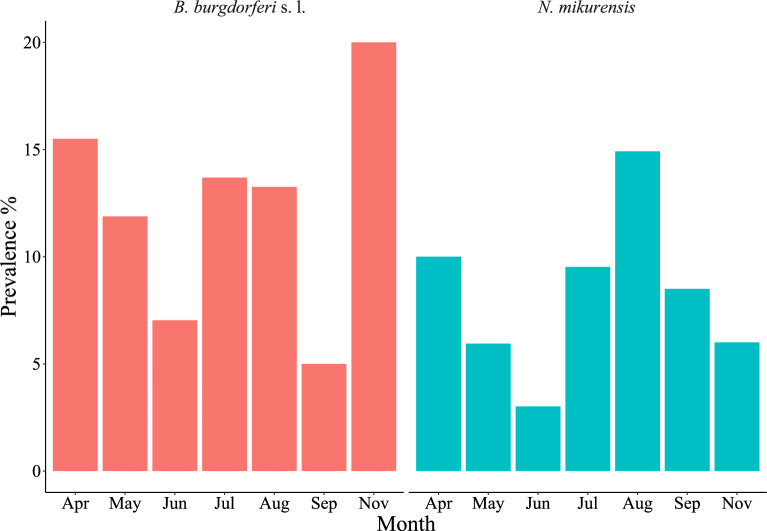
Monthly prevalence of *Borrelia burgdorferi* sensu lato and *Neoehrlichia mikurensis* in Mandal, southern Norway in 2020

The DON was highest in September with 44 nymphs and lowest in November with 8 nymphs, with an average of 27 nymphs (Table 3). A total of 5790 nymphs were collected, 4440 were selected for the surveillance of TBEV and 1350 for this study. All 1350 were confirmed as *I. ricinus*. No samples showed inhibition in the spike-in test. In total, *B. burgdorferi* s. l. was detected in 12% of samples (166/1350) and *N. mikurensis* in 8% (110/1350), including 2.3% (31/1350) coinfected ticks (Table 3). Coinfection was found between *B. afzelii* and *N. mikurensis* and *B. burgdorferi* s. s. and *N. mikurensis*. The highest prevalence of *B. burgdorferi* s. l. (20%) was found in November and the lowest (5%) in September. For *N. mikurensis* the highest prevalence (15%) was found in August and the lowest (3%) in June (Fig. 1).

**Table 3:** Prevalence of *Borrelia burgdorferi* sensu lato and *Neoehrlichia mikurensis* and density assessment of questing and infected *Ixodes ricinus* nymphs

Month	n	DON	*Bb*sl ( +)	% (CI)	DIN *Bb*sl	*Nm* ( +)	% (CI)	DIN *Nm*
Apr	200	21.5	31	16 (11–21)	3.3	20	10 (7–15)	2.2
May	202	35.5	24	12 (8–17)	4.2	12	6 (3–10)	2.1
Jun	199	35	14	7 (4–11)	2.5	6	3 (1–6)	1.1
Jul	168	24.3	23	14 (9–20)	3.3	16	10 (6–15)	2.3
Aug	181	21	24	13 (9–19)	2.8	27	15 (10–21)	3.1
Sep	200	44	10	5 (3–9)	2.2	17	9 (5–13)	3.7
Nov	200	8	40	20 (15–26)	1.6	12	6 (3–10)	0.5
Total	1350	27	166	12 (11–14)	2.8	110	8 (7–10)	2.1

%: prevalence. CI: 95% confidence intervals; n: number of samples; *Nm*: *Neoehrlichia mikurensis*; *Bb*sl: *Borrelia burgdorferi* s. l.; DON: number of questing nymphs in 100 m^2^; DIN: Density of infected nymphs

A strong negative correlation was found between DON and the prevalence of *B. burgdorferi* s. l. (r = -0.94; *P*-value = 0.001). No significant correlation was found between the DON and prevalence of *N. mikurensis* (r = -0.25; *P*-value = 0.591), nor with the DIN of *B. burgdorferi* s. l. (r = 0.28; *P*-value = 0.593) and *N. mikurensis* (r = 0.53; *P*-value = 0.213).

**Fig. 2:**
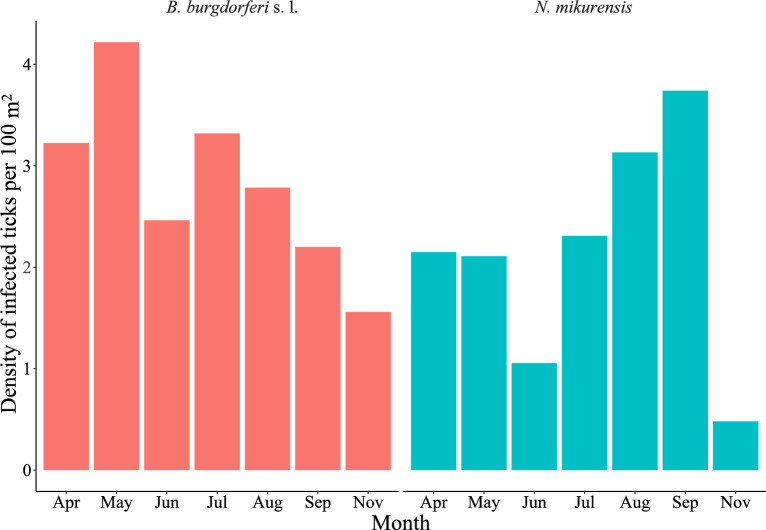
Monthly density of infected nymphs with *Borrelia burgdorferi* sensu lato and *Neoehrlichia mikurensis* in Mandal, southern Norway in 2020

For *B. burgdorferi* s. l. the DIN was highest in May (4.2) and lowest in November (1.6). For *N. mikurensis*, the DIN was highest in September (3.7) and lowest in November (0.5). The average DIN over the whole year was 2.8/100m^2^ for *B. burgdorferi* s. l. and 2.1/100m^2^ for *N. mikurensis* (Fig. 2, Table 3).

**Fig. 3:**
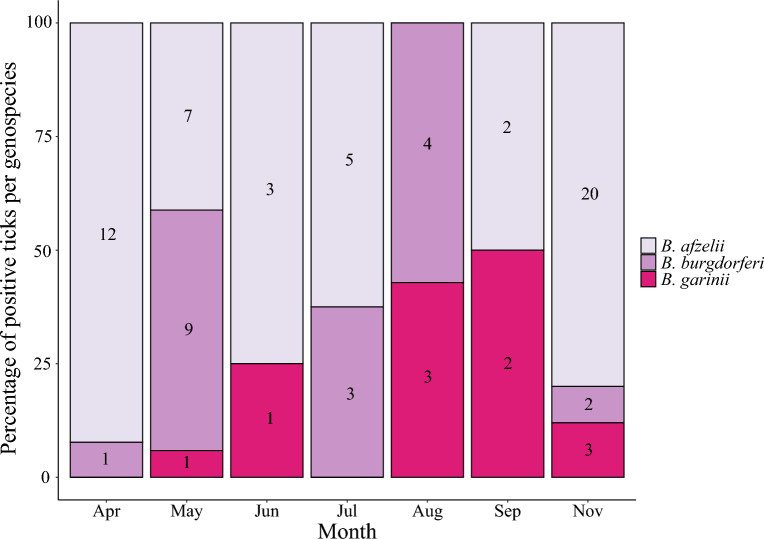
*Borrelia burgdorferi* sensu lato *g*enospecies distribution of positive samples per month in Mandal, southern Norway in 2020

Sanger-sequencing gave sequences of satisfactory quality in 47% of the samples (78/166 samples); 63% (49/78) were *B. afzelii*, 24% were *B. burgdorferi* s. s. (19/78), and 13% were *B. garinii* (10/78) (Fig. 3). Sequences are available in GenBank: accession numbers: PQ659299- PQ659373. The remaining 53% of samples were still considered true positives since they were positive in the two different PCRs targeting *B. burgdorferi* s. l.

## Discussion

In this study, the overall prevalence of *Borrelia burgdorferi* s. l. and *Neoehrlichia mikurensis* was 12% and 8%, respectively. These findings align with previous studies conducted in southern Norway [33] and are consistent with the reported ranges across Europe [17, 34]. Similar to earlier research in southern Norway [6, 17, 35], we observed a coinfection rate of 2%, which included combinations of *N. mikurensis* with *B. afzelii* and *N. mikurensis* with *B. burgdorferi* s. s., but not of *N. mikurensis* and *B. garinii*.

A marked increase in the prevalence of *B. burgdorferi* s. l. was observed from September to November. If *I. ricinus* nymphs in southern Norway present a bimodal phenology, the active nymphs in November are likely to belong to a younger cohort than ticks active in the spring and summer months [36]. This could imply that November ticks have fed as larvae in the spring and molted to nymphs during summer or early autumn. Under laboratory conditions, it has been demonstrated that the *B. afzelii* load in ticks decreases dramatically with time after the larvae to nymph molt [37]. This might increase the odds of detecting the bacterium by qPCR in recently molted ticks, as might be the case of ticks collected during November.

The prevalence of *B. burgdorferi* s. l. showed a strong negative correlation with the DON. In months with high tick density at the time of sampling, a lower proportion of the ticks were infected. However, infected nymphs were more active than uninfected nymphs during months with less tick density. Experimental studies suggest that *Borrelia*-infected ticks may exhibit reduced motor activity, remaining high in the vegetation, compared to uninfected ticks that return to the ground, leading infected ticks to prolonged questing periods under unfavorable conditions [38, 39]. If uninfected ticks avoid questing during unfavorable conditions, but infected ticks keep questing longer, the chance of collecting infected ticks would increase inversely proportional to the number of uninfected questing ticks.

A different seasonal pattern in the prevalence of *B. burgdorferi* s. l. was observed in 2007 in the same location in Mandal, Norway. The prevalence peaked at 38.2% in August and the lowest prevalence was 6.3% in April [11]. However, only 205 nymphs were analyzed in total, with the highest monthly prevalence estimation based on the analysis of just 34 ticks, and DON was not calculated. To provide a more robust estimation of prevalence, an average of 193 nymphs were analyzed per month in 2020, and tick population densities were assessed at each sampling.

Yearly variations in environmental conditions could also influence prevalence. For instance, a high rodent population may lead to higher prevalence of *B. burgdorferi* s. l. in ticks [20, 40]. Also, the years 2019–2020 in Mandal were characterized by higher precipitation and lower temperatures compared to 2006–2007 (data not shown). Variations in temperature and saturation deficit have been linked to the synchronization of larval and nymphal activity in *Ixodes* spp., which can positively influence the prevalence *B. burgdorferi* in the next generation of nymphs by cofeeding [41, 42]. Additionally, official hunting statistics suggest an increase in the density of cervids in Mandal since 2017, compared to previous years (Statistics Norway, www.ssb.no/). Cervids, including moose, red and roe deer, are known as incompetent hosts for *B. burgdorferi* s. l., reflected in reduced infection rates in questing *I. ricinus* in areas with high-deer densities compared to low-deer density areas [20, 43].

In nymphs collected across Europe, *B. afzelii* was the most common genospecies, followed by *B. garinii*, *B. valaisiana*, and *B. burgdorferi* s. s. [34]. Previous research in Mandal found *B. garinii* to be the dominant genospecies, followed by *B. afzelii*, *B. burgdorferi* s. s., and *B. valaisiana* [11]. In our study, *B. afzelii* was the most prevalent genospecies. However, as only 47% of the positive samples were successfully sequenced, this could introduce bias. As *B. afzelii* is primarily amplified by rodents and *B. garinii* by birds [20, 44], the low prevalence of bird-associated genospecies and the high prevalence of *N. mikurensis* suggest that birds contributed less significantly to the tick population during the study year in this site. In support of this hypothesis, very few ground feeding birds were observed during the samplings, but many tracks of rodents and deer were observed.

The density of infected nymphs with *N. mikurensis* and *B. afzelii* are positively dependent on rodent density, which, in turn, is driven by food availability for rodents [45]. In northern Scandinavia, rodents’ abundance follows cycles of approximately four years [46]. Similarly, cervid migration patterns vary by region in Norway, with snow cover being a primary driver of migration, an important factor in tick density [47, 48]. Given the relevance of the density of infected nymphs in the assessment of human infection risk, a multi-year approach in several sites is necessary to fully understand these ecological cycles [49–51].

## Conclusion

This study provides valuable insights and complements information into the variability in prevalence of *B. burgdorferi* s. l. and *N. mikurensis* in questing nymphal ticks in Mandal. This is the first monthly evaluation of the prevalence of *N. mikurensis* in ticks in Norway, showing a variation from 3 to 15% within the same year. Similarly, *B. borrelia* s. l. presented a variation in prevalence from 5 to 20%, contrasting with higher prevalences estimated in a previous study in the same locality. Furthermore, these findings underscore the importance of evaluating both pathogen prevalence and tick density in the light of human infection risk assessments. The dynamics of these bacteria and their tick vectors are closely tied to the population dynamics of vertebrate hosts, highlighting the need for long-term studies that incorporate tick, bird, rodent, shrew, and ungulate population surveillance alongside monitoring meteorological variables.

## Data Availability

DNA sequences of *B. burgdorferi* s. l. are available in GenBank database under the accession numbers PQ659299- PQ659373. The datasets used and/or analyzed during the current study are available from the corresponding author on reasonable request.

## Abbreviations

DIN: Density of infected nymphs
DNA: Deoxyribonucleic acid
DON: Density of nymphs

## Declarations

### Prior publication

Data included in this article have previously been published in the Ticks and tick-borne diseases conference, Weimar 2023; and in the Tick and Tick-borne pathogens Conference, Havanna-Varadero, 2024.
